# Recent Advances to Augment NK Cell Cancer Immunotherapy Using Nanoparticles

**DOI:** 10.3390/pharmaceutics13040525

**Published:** 2021-04-09

**Authors:** Kwang-Soo Kim, Dong-Hwan Kim, Dong-Hyun Kim

**Affiliations:** 1 Department of Radiology, Feinberg School of Medicine, Northwestern University, Chicago, IL 60611, USA; kskim@northwestern.edu; 2 School of Chemical Engineering, Sungkyunkwan University (SKKU), Suwon 16419, Korea; 3 Department of Biomedical Engineering, McCormick School of Engineering, Evanston, IL 60208, USA; 4 Robert H. Lurie Comprehensive Cancer Center, Chicago, IL 60611, USA; 5 Department of Bioengineering, University of Illinois at Chicago, Chicago, IL 60607, USA

**Keywords:** nanoparticles, NK cell therapy, cancer immunotherapy, tumor microenvironment, NK cell activation

## Abstract

Among various immunotherapies, natural killer (NK) cell cancer immunotherapy using adoptive transfer of NK cells takes a unique position by targeting tumor cells that evade the host immune surveillance. As the first-line innate effector cell, it has been revealed that NK cells have distinct mechanisms to both eliminate cancer cells directly and amplify the anticancer immune system. Over the last 40 years, NK cell cancer immunotherapy has shown encouraging reports in pre-clinic and clinic settings. In total, 288 clinical trials are investigating various NK cell immunotherapies to treat hematologic and solid malignancies in 2021. However, the clinical outcomes are unsatisfying, with remained challenges. The major limitation is attributed to the immune-suppressive tumor microenvironment (TME), low activity of NK cells, inadequate homing of NK cells, and limited contact frequency of NK cells with tumor cells. Innovative strategies to promote the cytolytic activity, durable persistence, activation, and tumor-infiltration of NK cells are required to advance NK cell cancer immunotherapy. As maturing nanotechnology and nanomedicine for clinical applications, there is a greater opportunity to augment NK cell therapeutic efficacy for the treatment of cancers. Active molecules/cytokine delivery, imaging, and physicochemical properties of nanoparticles are well equipped to overcome the challenges of NK cell cancer immunotherapy. Here, we discuss recent clinical trials of NK cell cancer immunotherapy, NK cell cancer immunotherapy challenges, and advances of nanoparticle-mediated NK cell therapeutic efficacy augmentation.

## 1. Introduction

Cancer is the second cause of death worldwide and still needs much effort to cure the desperate disease [1]. In the past decade, immunotherapies modulating anticancer immune responses for cancer elimination have made a remarkable revolution in cancer treatments [2]. The 2018 Nobel Prize in Physiology or Medicine selected immunotherapy pioneers. More understanding of immune system-related cancer biology at the cellular and molecular levels has allowed cancer immunotherapy to be rapidly advanced for clinical applications. Subsequently, various cancer immunotherapies, including immune checkpoint blockades, chimeric antigen receptor (CAR) T-cell therapy, cytokine therapy, natural killer (NK) cell therapy, and cancer vaccines, are able to exhibit notable successes in the clinics [3,4,5,6,7,8,9]. However, immune-suppressive tumor microenvironment (TME), immuno-therapeutic resistance, immuno-therapeutic ignorance, and off-target toxicity, i.e., immune-related adverse effects (irAEs), permitted only a small percentage of patients to experience a positive response [10,11,12]. Substantial effort to overcome the limitation of cancer immunotherapy and further development of cancer immunotherapy strategies are needed to advance this effective strategy to treat cancers [13].

**Figure 1:**
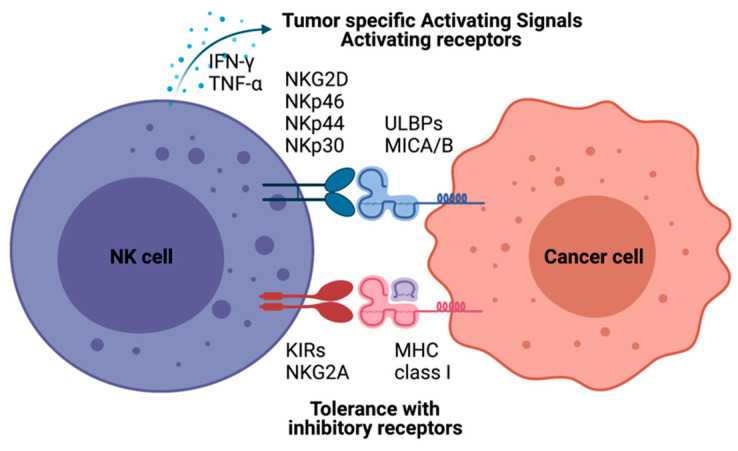
Natural killer (NK)-cell-mediated cytotoxicity. NK cell recognizes cells with NK cell receptors. MHC class I on target cells plays an inhibitory role binding to KIRs and NKG2A molecules resulting tolerance of NK cells as “self-recognition”. Otherwise, malignant cells inducing MICA/B, UL16 binding proteins (ULBPs), are detected by NK cells with NK-cell-activating receptors, including KG2D and NKRs. NK cells also have immune modulatory functions by producing IFN-γ and TNF-α to recruit other immune cells, such as dendritic cells and T cells.

NK cell cancer immunotherapy using adoptive transfer of NK cells takes a unique position to target tumor cells that evade the host immune surveillance [14,15,16]. NK cells belonging to innate lymphoid cells are cytotoxic, play roles in producing cytokines and essential immunosurveillance for viral infection and cancers [17,18]. NK cells are mainly localized at epithelial surfaces and quickly responding to pathogen invasion to maintain tissue homeostasis [19]. The lack of antigen receptors in NK cells is different from T and B lymphocytes. A wide range of germline-encoded activating and inhibitory receptors of NK cells can be engaged by particular ligands displayed on various kinds of cells (Figure 1). NK cell’s selective cytotoxic functions killing disease cells are finely tuned by the signaling balance between the activating and inhibitory receptors [20]. For healthy cells, NK cells preserve tolerance towards surrounding normal cells. The tolerance is mainly controlled through inhibitory receptors such as killer immunoglobulin-like receptors (KIRs) and natural killer group 2A (NKG2A) recognizing self-major histocompatibility complex (MHC) class I molecules (Figure 1) [21]. In the process of NK cell education, the strength of these inhibitory receptor/ligand interactions also strongly correlates with the generation of functional NK cells. The activation of the “turn on” signal for the selective cytotoxic effect is involving several activation receptors, including natural cytotoxicity receptors (NCRs: NKp46, NKp44, and NKp30) and natural killer group 2D (NKG2D), whose ligands are mainly stress-inducible molecules UL16 binding proteins (ULBPs), MHC class I chain-related protein A and B (MICA/B). With the activation receptors, NK cells can selectively attack virally infected cells or cancer cells that are expressing downregulated MHC class I molecules through “missing self-recognition” and “induced self-recognition” (Figure 1) [22]. 

These well-orchestrated selective cytotoxic functions of NK cells have prompted their use in many clinical trials to control tumor growth via their effector capacity. NK cell cancer immunotherapy has been considered an effective cancer treatment and a potent adjuvant to standard cancer treatment [23]. A total of 288 clinical trials are investigating NK cell immunotherapies to treat hematologic and solid malignancies in 2021 (www.clinicaltrials.gov). Those clinical trials using autologous NK cells, allogeneic NK cells, NK cell lines, and genetically modified NK cells have shown encouraging results in the response rate for various malignancies [24,25,26]. However, there are still considerable challenges in NK cell therapy to treat cancer patients. The TME structure and altered tumor immunogenicity lead to functional damage of NK cells and poor tumor trafficking and infiltration of NK cells into tumors [27]. Thus, various strategies to promote the expansion, cytolytic activity, durable persistence, activation, and tumor-infiltration of NK cells have been studied [28,29,30,31]. Recently, various multifunctional nanoparticles have been suggested to augment NK cell therapy for the treatment of cancers [32,33]. Active molecules/cytokine delivery, imaging, and physicochemical properties of nanomaterials are well equipped to overcome NK cell cancer immunotherapy challenges [34,35]. As maturing nanotechnology and nanomedicine for the clinical applications, there is greater opportunity for NK cell cancer immunotherapy. Here, we discuss recent NK cell adoptive cell transfer (ACT) clinical trials, challenges, and advances of nanoparticle-mediated NK cell therapeutic efficacy augmentation. 

## 2. NK Cell Cancer Immunotherapy Clinical Trials

**Figure 2:**
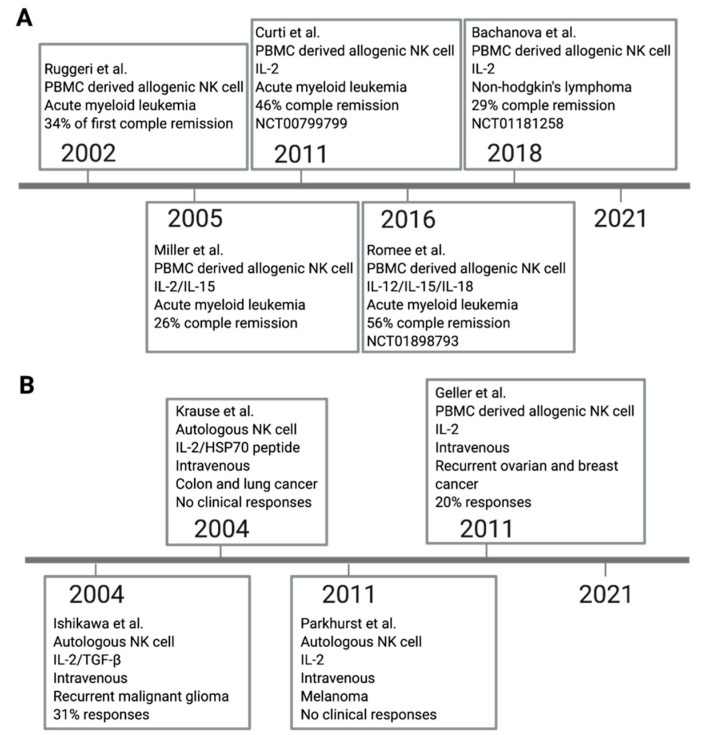
Clinical trials of NK-cell-based cancer therapy. A large number of NK cell clinical trials have been studied, and some remarkable studies in hematologic cancers (**A**) and solid tumors (**B**) were selected.

Adoptive transfer of ex vivo expanded autologous NK cells have been examined for the treatment of patients with lymphoma, colon cancer, breast cancer, and lung cancer in clinical trials (Figure 2A). The first remarkable clinical benefit of NK cell adoptive transfer was reported in 2002. KIR ligand mismatched alloreactive NK cells from peripheral blood mononuclear cells (PBMC) were applied to acute myeloid leukemia (AML) patients with human leukocyte antigen (HLA) mismatch hematopoietic transplantation. The first results showed 34% complete remission without graft versus host disease (GVHD) [36]. Later in 2005, IL-2 and IL-15 were added to ex vivo activated allogeneic KIR/KIR ligand mismatch PBMC derived NK cells were treated for AML patients [16]. Subsequently, alloreactive PBMC derived NK cells have been more investigated as immunotherapy of hematologic malignancies. Several results in phase I and II trials have demonstrated that allogeneic NK cell adoptive transfer has a clinical benefit with low severe adverse effects [16,36]. In 2011, a phase I trial showed IL-2 pre-activated NK cells from allogeneic KIR/KIR ligand mismatch donors were effective in highly-risk AML. In total, 6/13 patients obtained complete remission (NCT00799799) [37]. Another phase I trial reported that IL-12, IL-15, and IL-18 induced memory like allogeneic NK cells demonstrated robust responses against myeloid leukemia with 5/9 clinical responses including four complete remissions (NCT01898793) [24]. Then, the latest phase II clinical trial tested the efficacy of allogeneic haploidentical NK cells. It demonstrated only 29% (4/14) had objective responses in non-Hodgkin lymphoma patients (NCT01181258) [25]. The outcomes of NK cell therapy are still limited compared to other therapeutic options. 

The initial successes of adoptive NK cell transfer in treating hematological cancers also prompted clinical endeavors in using the strategy against solid cancers (Figure 2B). Unfortunately, the clinical efficacy of NK cell transfer to the solid tumor has been disappointing [27]. One of the clinical trials for solid tumor treatment is autologous NK cell therapy. Ex vivo expanded NK cells with IL-2 and IFN-β were used for the malignant glioma patients, but the systemically applied NK cell cancer immunotherapy resulted in only 5/16 responses [38]. In another study, pre-treated autologous NK cells with IL-2 and HSP70 had been infused for colorectal or non-small-cell lung solid tumor patients. Immunological responses were observed, but no clinical treatment-related response was reported [39]. More recent clinical trials of IL-2 activated autologous NK cells evaluated their efficacy for metastatic melanoma and renal cell carcinoma patients. However, the response rate was 0% (0/8 responses) [15]. Another phase II trial used allogeneic NK cells for recurrent ovarian and breast cancer patients. IV injected IL-2 activated allogeneic haploidentical NK cell adoptive transfer showed the partial response of 4/20 response [40]. 

With the limited response of NK cells in solid tumors, current clinical trials of NK cell therapy have more focused on the combination therapy with NK cells and chemotherapy (Table 1). A combination of NK cell therapy with 5-Fu and oxaliplatin was tested for advanced colon carcinoma patients. The 5-year progression-free survival and overall survival rates in the combination NK cell therapy group were significantly higher than those in the control group of pure chemotherapy (51.1% versus 35%, *p* = 0.044; 72.5% versus 51.6%, *p* = 0.037, respectively) [41]. However, another phase I clinical trial results of adoptive transfer of IL-2 activated autologous NK cells in combination with IgG1 antibody (Trastuzumab or cetuximab) showed poor response rate in gastric or colorectal cancer patients (0/6 responses) [42]. Despite these various endeavors for NK cell cancer immunotherapy, such clinical responses have been diverse and not been robust as it has been expected.

## 3. Challenges of NK Cell Cancer Immunotherapy

**Figure 3:**
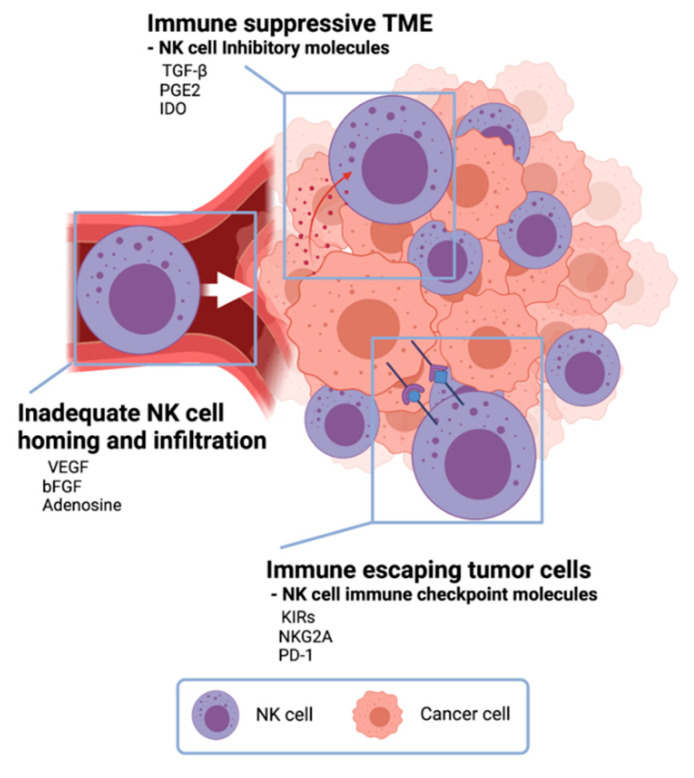
Challenges in NK cell cancer immunotherapy. In the tumor microenvironment, cancer cells secrete anti-immune molecules TGF- β, PGE2, and indoleamine 2,3-dioxygenase (IDO) to evade NK-cell-mediated tumor-cell lysis. Vascular endothelial growth factor (VEGF), basic fibroblast growth factor (bFGF), and adenosine inhibit NK cells from homing to tumors resulting inadequate NK cell infiltration in tumors. Tumor cells express NK cell immune checkpoint molecules to escape from NK cells.

The primary reason for the therapeutic limitation of NK cell cancer immunotherapy was attributed to the immune-suppressive TME, low activity of NK cells, inadequate homing of NK cell adoptive transfer, and limited contact frequency of NK cells with tumor cells (Figure 3) [23,43].

One of NK cell therapy’s limitations is the immunosuppressive effect of the TME (Figure 3) [6,44]. TME has unique environments built with various cancer cell-derived cytokines and following abnormal metabolic profiles. NK cells in the TME are changed to be low proliferation, decreased cytokine release, and downregulation of activation receptors [27]. Especially, there are immune-suppressive TME cytokines such as TGF-β, prostaglandin E2 (PGE2), and indoleamine 2,3-dioxygenase (IDO) [45]. Along with immune suppressive cytokines from tumor cells, regulatory T cells and myeloid-derived suppressor cells usually inhibit both the expansion and the function of effector NK cells with downregulating NK cell-activating receptors, IFN-γ, and cytolytic molecules [46]. To overcome this immune-suppressive TME, TME modulation involved with TGF-β, PGE2, and IDO cytokines and NK cell activation using IL-2, IL-12, IL-18, and IFN-γ have been actively studied for the augmentation of NK cell cytotoxicity against tumor cells in TME [47].

Tumors also exploit several defense mechanisms to limit NK cell homing and infiltration [48]. Deregulation of chemokine expression in the tumor is an important mechanism preventing NK cell infiltration and homing (Figure 3). Vascular endothelial growth factor (VEGF) and basic fibroblast growth factor (bFGF) signaling on endothelial cells can repress adhesion molecule expression and prevent NK-cell infiltration [49,50]. So, there are many ongoing efforts toward improving tumor infiltration of adoptively transferred NK cells. The modification of NK cells with tumor-specific molecules and chemokine-chemokine receptor axis has been tried [51]. CCR5-CCL5 axis was induced to enhance NK cell infiltration in tumor tissue, and CXCR3 on NK cells also could interact with CXCL9, CXCL10, and CXCL11 from tumor cells [52,53]. The local delivery of those modified NK cells and catalytic molecules for migration was also studied to enhance NK cell therapy approaches. Ultrasound-mediated-, magnetic field-mediated-, and catheter-directed-NK cell delivery have demonstrated improved NK cell homing and infiltration [54,55,56]. 

Another critical challenge of NK cell adoptive transfer is the immune escape of tumor cells (Figure 3) [6,27]. Mutated tumor cells are expressing immune checkpoint molecules, rendering the immune system to be ineffective. Although the NK cell activation process involves more than one receptor–ligand interaction, NK-cell-mediated anticancer efficacy is often hindered by the low expression of NK cell activation receptor–ligand [57]. Subsequently, NK cell recognition in the tumor site is hampered due to the lack of NK–tumor contact. Highly effective immune cell engagers and specifically designed receptors effectively enhance the recognition and contact of NK cell-activating receptors [58]. Recently, CAR expressed NK cells showed an efficient cancer cell killing effect in CD19 positive leukemia and lymphoma cells [26]. Anti-CD19 CAR T cells were approved by the US Food and Drug Administration (FDA) in 2017. Despite the robust clinical response, the severe adverse effect was recognized [59]. Further studies for preventing immune escaping tumor cells are needed.

## 4. Nanomaterials for NK Cell Cancer-Immunotherapy

**Figure 4:**
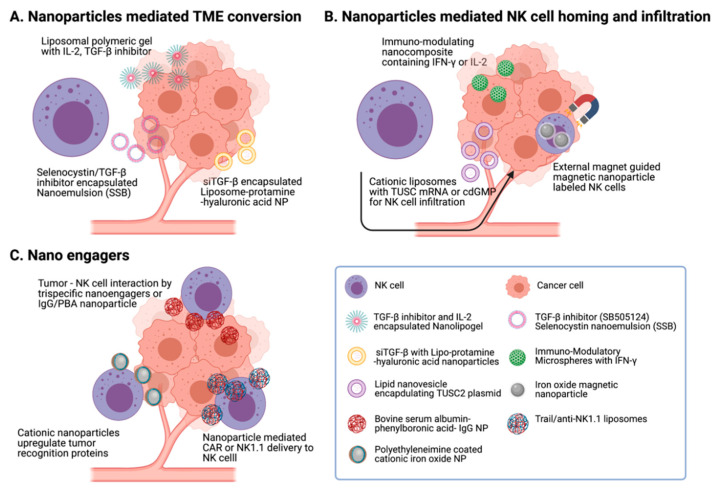
Nanomaterials for NK cell cancer-immunotherapy. Various nanoparticle-mediated strategies have been developed to augment NK cell therapeutic efficacy. (**A**) Liposome and nano-emulsion were used to modify tumor microenvironment with the inhibition of TGF-β signals. (**B**) Nanoparticles or liposomes encapsulating IFN-γ or tumor suppressor candidate (TUSC) improve NK cell homing. Magnetic nanoparticles labeled NK cells were guided into the tumor site, using external magnetic field applications. (**C**) Nanoengagers that enhance NK cells’ tumor recognition reinforced NK-cell-mediated tumor-cell-killing efficacy.

Nanomaterial functionality, including various payloads delivery, reactivity with cellular ligands, and imaging contrast, facilitates effective chemical or biological modification of cells and cancer therapeutic applications [34,60,61,62,63]. Versatile multifunctionality of nanomaterials and their compatibility with NK cells also have a great potential to boost NK cell function in the ex vivo expansion of NK cells, activation of NK cells, and intra/post-operative procedures of NK cell therapy (Figure 4). Recent reports demonstrating augmented NK cell cancer immunotherapy using nanoparticles are summarized here (Table 2). 

### 4.1. Nanoparticle-Mediated Conversion of Immune Suppressive TME for NK Cell Cancer Immunotherapy

The vitality of NK cells is strongly influenced by tumor secreted TGF-β. Particularly, TGF-β is a negative regulator of IFN-γ production and downregulates the surface expression of the NK cell-activating receptors such as NKG2D, NKp46, and NKp30, causing reduction of the cytotoxic activity and anticancer function of NK cells. Thus, there were many attempts to moderate the immune-suppressive TGF-β signaling in TME. In 2014, Xu et al. reported a strategy to manipulate TGF-β signaling with lipid calcium phosphate nanoparticles and liposome protamine hyaluronic acid nanoparticles. Successfully delivered TGF-β siRNA downregulated ~50% TGF-β in TME. It resulted in increased NK cell infiltration and decreased level of regulatory T cells in the melanoma model [64]. Another revolutionary research using liposomal polymeric gels showed the reversed antitumor activity of NK cells in the immune suppressive TME. TGF-β inhibitor and IL-2 were co-delivered with a liposome polymeric gel into the tumor site. Significant tumor inhibition and enhanced survival were demonstrated in melanoma and triple negative breast cancer-bearing mice [65]. Recently, Chang Liu et al. developed nano-emulsion with selenocysteine and TGF-β antagonists. The local treatment of nano-emulsion reduced the side effects of TGF-β antagonists and effectively suppressed TGF-β/TGF-βRI/Smad2/3 signaling. Subsequently, the locally delivered TGF-β antagonists reinforced the upregulation of NKG2DLs on cancer cells [76]. Various bio-nanoparticle formulations, which can modulate immune-suppressive TME, are expected to be a novel tool for supporting NK cell cancer immunotherapy.

### 4.2. Nanoparticle-Mediated NK Cell Homing and Infiltration

In the past few decades, CXCL/CXCR axis and cytokines such as IL-2 and IL-15 were regarded as appropriate options to improve NK cell homing and infiltration to tumor sites [52,66]. Recent nanotechnology further enables developing a diverse spectrum of strategies to achieve better homing properties of NK cells. NK cell homing and infiltration are strongly related to the efficacy of NK cell cancer immunotherapy. Recently, various nanoparticle-mediated approaches showed a promise to enhance NK cell tumor infiltration for NK cell cancer immunotherapy. A study utilized chitosan-based nanoparticles to deliver a fused dsNKG2D–IL-21 gene to the tumor region. The delivered DNA fragments encoding NKG2D ligand increased secretion of the NKG2D ligand and IL-21, resulting in NK cell infiltration and enhanced NK cell and T cell activation in the tumor tissue. Intravenous administration of dsNKG2D–IL-21-chitosan nanoparticles considerably delayed tumor growth and prolonged the life span of treated mice [67]. Another report by Meraz et al. developed tumor suppressor candidate 2 (TUSC2) plasmid DNA-loaded cationic liposomes to investigate its potential for NK cell activation and the increase of NK cell population in the TME. TUSC2 is one of the strong tumor suppressor genes to control various kinds of tumors. However, TUCS2 mRNA is commonly downregulated or suppressed in TME and resulted in low overall survival rate. In this research, the systemic delivery of TUSC2 nanovesicle converted gene expression profile in TME and resulted in immune effector NK cell infiltration for a survival benefit in the preclinical model [68]. In the same context of nanoparticle-mediated cytokine treatment facilitating NK homing and infiltration to tumors, a PEGylated liposome was used to deliver various TLR-4 agonists, such as cdGMP, a STING agonist, and MPLA for inducing type I interferons in the TME [69]. Another study used nanocomposite to enhance tumor-specific migration on NK cells via modulating chemokine signaling. Park et al. developed immunomodulatory nanocomposite microspheres containing recombinant IFN-γ. Their delivery of IFN-γ eluting nanocomposite microspheres induced a significant increase of NK cell infiltration. This strategy showed improved accumulation and retention of NK in tumors, resulting in significantly enhanced therapeutic efficacy [70]. Additionally, NK cell guiding IL-2 [23] was delivered with nanoparticles. Zhang et al. reported that liposome-anchored IL-2/anti-CD137 achieved antitumor activity without systemic toxicity of IL-2 [77]. Frick et al. also demonstrated the enhanced in vivo targeting of NK cells with starch nano-capsules coupled with IL-2 [78,79].

As another approach, external magnetic-field-responsive magnetic nanoparticles were used to guide NK cells to the tumor. Jang et al. developed fluorophore Cyanine 5.5-silica-decorated superparamagnetic iron oxide nanoparticles and conjugated with NK cells. The magnetic nanoparticle-conjugated NK cells were intravenously injected into B cell lymphoma-bearing mice, and a neodymium magnet (340 G/mm) was put adjacently to the tumor site. Consequently, NK cell infiltration in tumors was increased by 17-fold when applying the magnetic field [71]. Wu et al. also used polydopamine-coated iron oxide nanoparticles and an implanted plate magnet in the hypodermic tumor. The injection of magnetic nanoparticles loaded NK cells significantly improved the accumulation and retention of NK in tumors [55]. 

### 4.3. Nanoparticle-Mediated NK Cell Receptor-Ligand Activation and Nano-Engagers

Another strategy for robust tumor regression by NK cells is to upregulate the NK cell-activating responses and trigger the production of NK cell stimulatory cytokines in the TME. Direct interaction between NK cells and tumor cells has been shown to coordinate adaptive immunity by recruiting dendritic cells and T cells [80]. Chandrasekaran et al. adorned liposomes with TRAIL and anti-NK1.1 proteins via maleimide-thiol chemistry, and the liposomes were conjugated with NK cells. The nano-liposome conjugated NK cells prolonged their presence within the tumor-draining lymph nodes, and continuous NK cell activation effectively inhibited the spread of cancer cells from the primary site [72]. Another novel strategy used cationic nanoparticles to improve NK cell activation. A simple and efficient method using polyethyleneimine coated-cationic iron oxide nanoparticles enhanced over 2-fold higher cytotoxicity against triple negative breast cancer. Cationic nanoparticle-treated NK cells expressed higher CCR4 and CXCR4 and showed better interaction with breast cancer cells [73]. Zheng et al. successfully modified the tumor cell surface with rationally designed immunomodulating nanoparticles. Nanoparticles were composed of bovine serum albumin nano-capsule core and Immunoglobulin G (IgG)/phenylboronic acid (PBA) shell structure for enhancing NK cell (IgG) binding to tumor cell (PBA). In vivo study exhibited NK-cell-mediated direct conjugation to tumor cells and effective tumor growth inhibition [74]. As an advanced study, Bi- or Tri- specific targeting materials that can connect different types of cells and stimulate activating receptors on effecter cells were suggested. Au et al. fabricated PLGA nanoparticle-based tri-specific NK cell engagers that can target epidermal growth factor receptors on tumor cells and CD16+/4-1BB+ NK cells. This multi-specific nano-platform successfully allowed the NK cell engagement to tumor cells and NK cell activation to attack EGFR positive colorectal adenocarcinoma, triple negative breast cancer, epidermoid carcinoma, and melanoma, respectively [75]. Further, since remarkable success in CAR-T cell therapy, the engagement of immune effector NK cells to the tumor cells is one of the promising approaches in NK cell cancer immunotherapy. 

## 5. Conclusions and Future Outlook

NK-cell-based therapy has attracted significant attention in research on cancer treatment. Considering the tumor-specific cytotoxic function of NK cell cancer immunotherapy, NK cell cancer immunotherapy can be useful in many clinical cases. However, achieving meaningful therapeutic outcomes in the clinic is still challenging, owing to difficulties with the numerous immune-suppressive factors in the TME and poor NK cells’ homing and infiltration. Further development and refining of NK cell cancer immunotherapy are required. One promising direction would be the combinational NK cell cancer immunotherapy with other synergistic cancer therapies. Because NK cell infusion appeared to be safe and NK cells do not need a particular process of antigen recognition by antigen-presenting cells, there is the strength as a combination therapy regime. Utilizing distinct mechanisms of NK cells will be critical to have synergistic effects in combination with other cancer therapies, including chemotherapy, immune-modulating cytokines (IL-2 and IFN-γ) therapy, and immune checkpoint blockades immunotherapy. In advance of NK cell cancer therapy, nanoparticles will be a key tool, as they have recently shown great potential for augmenting the therapeutic efficacy of NK cell cancer immunotherapy. Engineered nanoparticles delivering various therapeutic agents, including antibodies, stimulatory cytokines, genes, or adjuvants, will enhance the NK cell activity, NK cell proliferation, and NK cell migration to tumor sites, thus markedly inhibiting tumor progression. The development of nanoparticles targeting the TME can also readily upregulate NK cell-activating ligands and stimulatory cytokines (Figure 4). Further, the medical imaging contrast effect of nanoparticles will allow image-guided NK cell cancer immunotherapy that can monitor NK cells and therapeutic prognosis. Indeed, the multifunctionality of nanoparticles interplaying between the immune system and tumors will allow the synergistic combinational anticancer effect, broaden the capacity of NK cell cancer immunotherapy, and contribute to developing safe and controlled NK cell cancer immunotherapies. 

## Figures and Tables

**Table 1:** Selected recent ongoing NK cell-based combination clinical trials.

Year	Cell Source	Pre-Treatment	Tumor Type	Combination	Ref.
2008	Autologous	IL-2	CML, Pancreatic cancer, Colorectal cancer, Multiple myeloma, Non-small cell lung cancer	Bortezomib	NCT00720785
2012	UCB Allogenic		Multiple Myeloma	Elotuzumab, Lenalidomide, Melphalan	NCT01729091
2013	PBMC Allogenic	IL-12, IL-15, IL-18	AML, MDS	ALT-803.	NCT01898793
2013	PBMC Allogenic		Neuroblastoma	Anti-GD2	NCT01857934
2014	Autologous	IL-2	HER2+ Breast and Gastric Cancer	Trastuzumab	NCT02030561
2015	Autologous	IL-2	Head and Neck Cancer	Cetuximab	NCT02507154
2016	PBMC Allogenic	IL-2	Neuroblastoma	Anti-GD2	NCT02650648
2016	PBMC Allogenic		Hematologic, solid cancers	ALT803	NCT02890758
2016	Autologous	IL-15	Multiple Myeloma	Elotuzumab	NCT03003728
2017	PBMC Allogenic	IL-15, GSK3beta inhibitor	Advanced solid tumors	Trastuzumab, Cetuximab	NCT03319459
2017	PBMC Allogenic	IL-15, GSK3beta inhibitor	Ovarian cancer	IL-2	NCT03213964
2017	UCB Allogenic		NHL	Rituximab	NCT03019640
2017	PBMC Allogenic	IL-2	Neuroblastoma Recurrent	Anti-GD2	NCT03242603
2018	UCB Allogenic		Relapsed or Refractory Solid Tumors	Cyclophosphamide, Etoposide	NCT03420963
2018	PBMC Allogenic	K562-mbIL15-41BBL.	Relapsed or Refractory Neuroblastoma	Anti-GD2	
2019	PBMC Allogenic		Recurrent Ovarian Carcinoma		

**Table 2:** Preclinical strategy of nanoparticle-based NK cell therapy.

Year	Cell Source	Nanoparticle	Tumor Type	Combination	Ref.
2014	Endogenous	Lipid-calcium-phosphate nanoparticle and liposome-protamine-hyaluronic acid nanoparticle	Melanoma	siTGF-β	[64]
2012	Endogenous	Liposomal polymeric gel	Metastatic melanoma	TGF-β inhibitor (SB505124)	[65]
2020	NK-92	Nanoemulsion	Triple negative breast cancer	Selenocysteine, TGF-β inhibitor (SB505124)	[66]
2017	Endogenous	Chitosan nanoparticle	Colon cancer	NKG2D, IL-21	[67]
2018	Endogenous	DOTAP:cholesterol nanovesicle	Lung cancer	TUSC2 gene, anti-PD-1	[68]
2019	Endogenous	Lipid nanoparticle	Triple negative breast cancer	cdGMP, monophosphoryl lipid A	[69]
2017	Endogenous	PLGA microsphere	Hepatocellular carcinoma	IFN-γ, Transcatheter intra-arterial infusion	[70]
2012	NK-92MI	Magnetic nanoparticle	B cell lymphoma	External magnetic field	[71]
2018	Human primary NK cell	Magnetic nanoparticle	Non-small cell lung cancer	External magnetic field	[55]
2015	Mouse primary NK cell	TRAIL-coated liposome	Lymph node metastatic cancer	TRAIL, anti-NK1.1	[72]
2020	NK-92MI	Cationic magnetic nanoparticle	Triple negative breast cancer	-	[73]
2019	Human primary NK cell	Immunomodulating nanoparticle	Triple negative breast cancer	phenylboronic acid, IgG	[74]
2020	Mouse primary NK cell	Trifunctional PLGA nanoparticle	EGFR positive solid tumor	Epirubicin	[75]

## Data Availability

Not applicable.
